# Supplementary material for: Repurposing the atypical type I-G CRISPR system for bacterial genome engineering
Source: Microbiology (Reading). 2023 Aug 1;169(8):001373. doi: 10.1099/mic.0.001373 (PMC10482374; doi:10.1099/mic.0.001373)
Supplement: Supplementary material 1 [file mic-169-1373-s001.pdf]

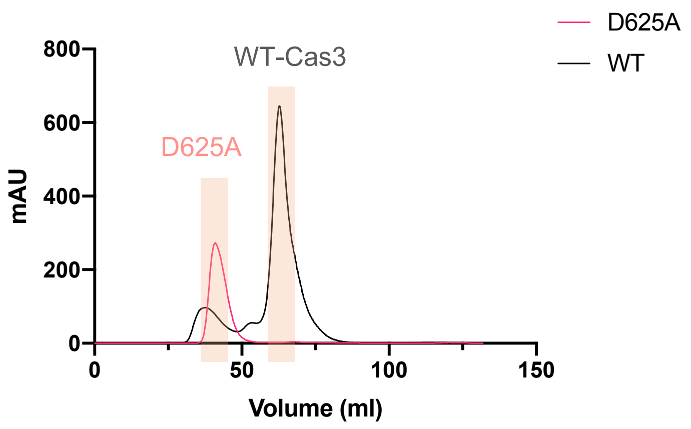

FigureS1. Cas3 D625A variant is aggregated. Size Exclusion chromatography of WT Cas3 and Cas3 D625A. Cas3 D625A eluted at right after void volume, suggesting an aggregated state.

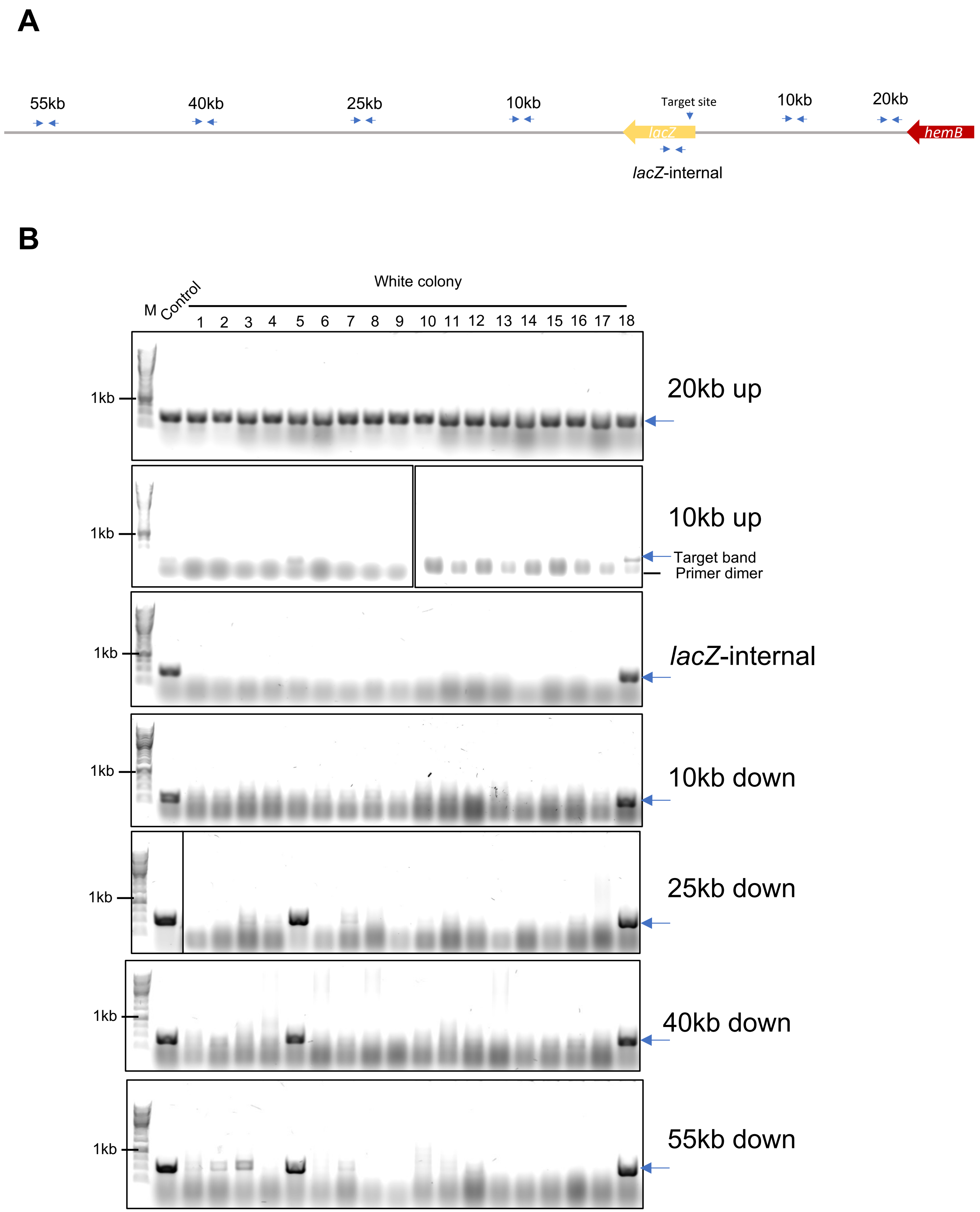

Figure S2. Tiling PCR of type I-G targeting *lacZ* . (A) An overview of location of tiling PCR primers; A pair of small blue arrow represents a set of PCR primers. **(B)** Tiling PCR product from different primers was submitted for electrophoresis on a 0.8% agarose gel. 18 white colonies and 1 blue colony (Control) were assayed. M, marker. Blue arrows indicate positive PCR products.

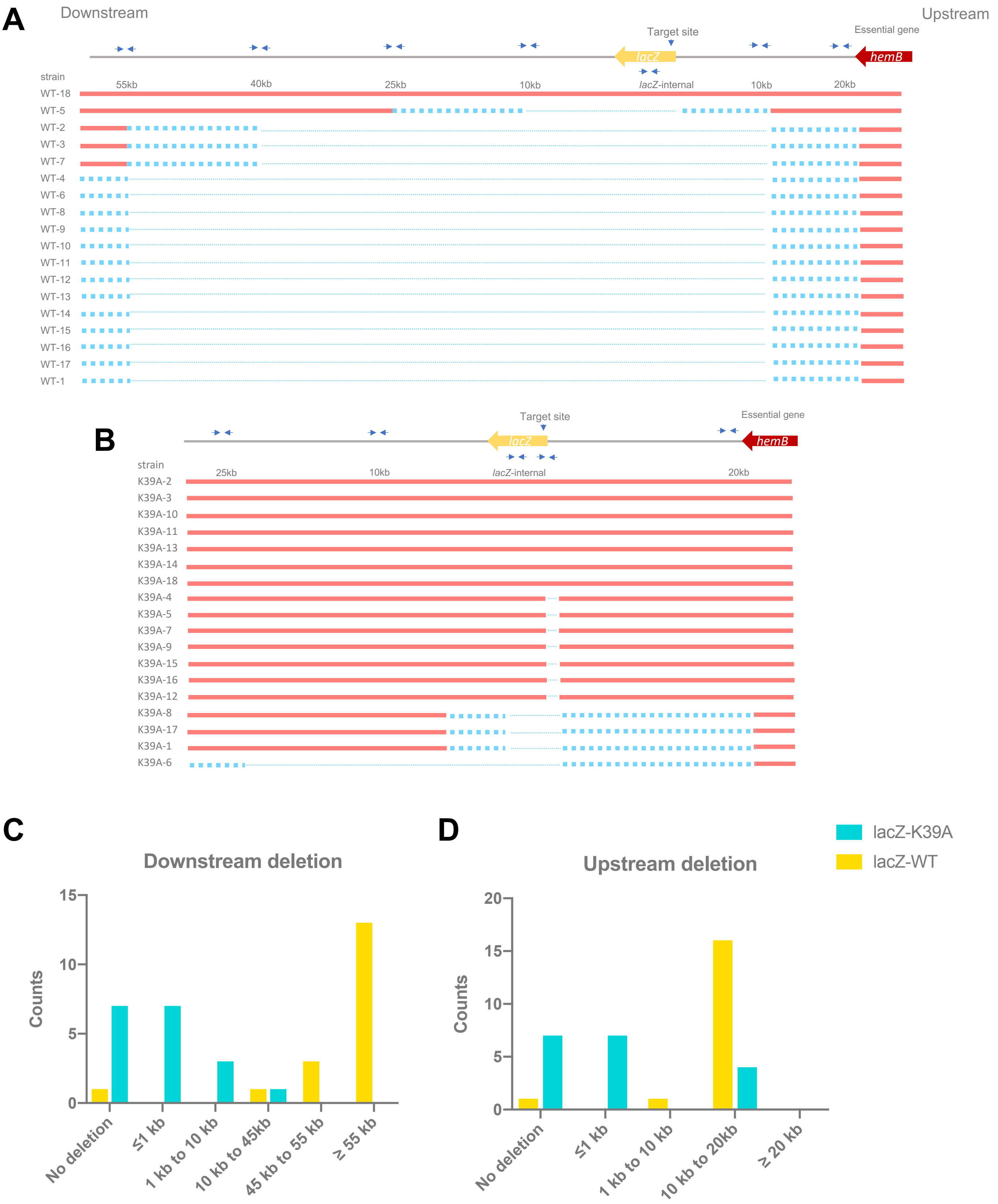

Figure S3. Type I-G editing on *lacZ*. (A) (B) A deletion map showing the outcome of *lacZ* targeting by wildtype type I-G or Cas3 K39A; 18 white colonies generated by *lacZ* targeting were submitted for tiling PCR to determine the deletion range; Pairs of small blue arrow represent tiling PCR primers; Red lines indicate the intact sequence on genome; Blue dot lines indicates possible deleted sequence; Thin blue dash lines indicate confirmed deleted sequence. **(C) (D)** Editing outcome of *lacZ* targeting by wildtype type I-G (yellow) or Cas3 K39A (green); 18 white colonies generated by *lacZ* targeting were submitted for tiling PCR to determine the deletion range; Counts of white colonies are plotted against deletion range.

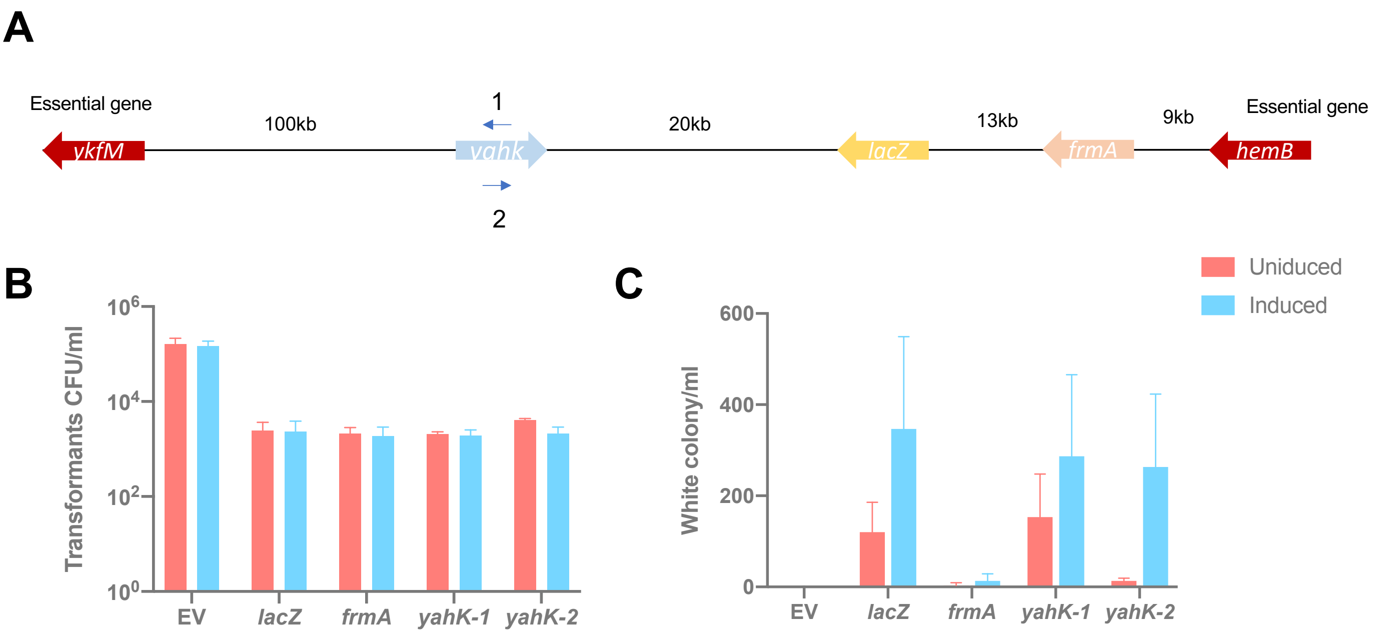

FigureS4. *yahK* and *frmA* target editing. (A) *yahK* and *frmA* location on *E.coli* genome. Arrow indicates direction of degradation with yahK-1 or yahK-2 spacer if the degradation is unidirectional.**(B) (C)** Transformants number and white colony number out of transformants on the plate after transformation of the empty vector, *lacZ* target spacer, *frmA* target spacer or *yahK* target spacer respectively with L-arabinose induction (blue) or without induction (red); Transformation efficiency was calculated as the number of transformants divided by the number of transformants for original plasmid without target (Empty vector control); Values and error bars represent the mean of three biological replicates and standard deviation.

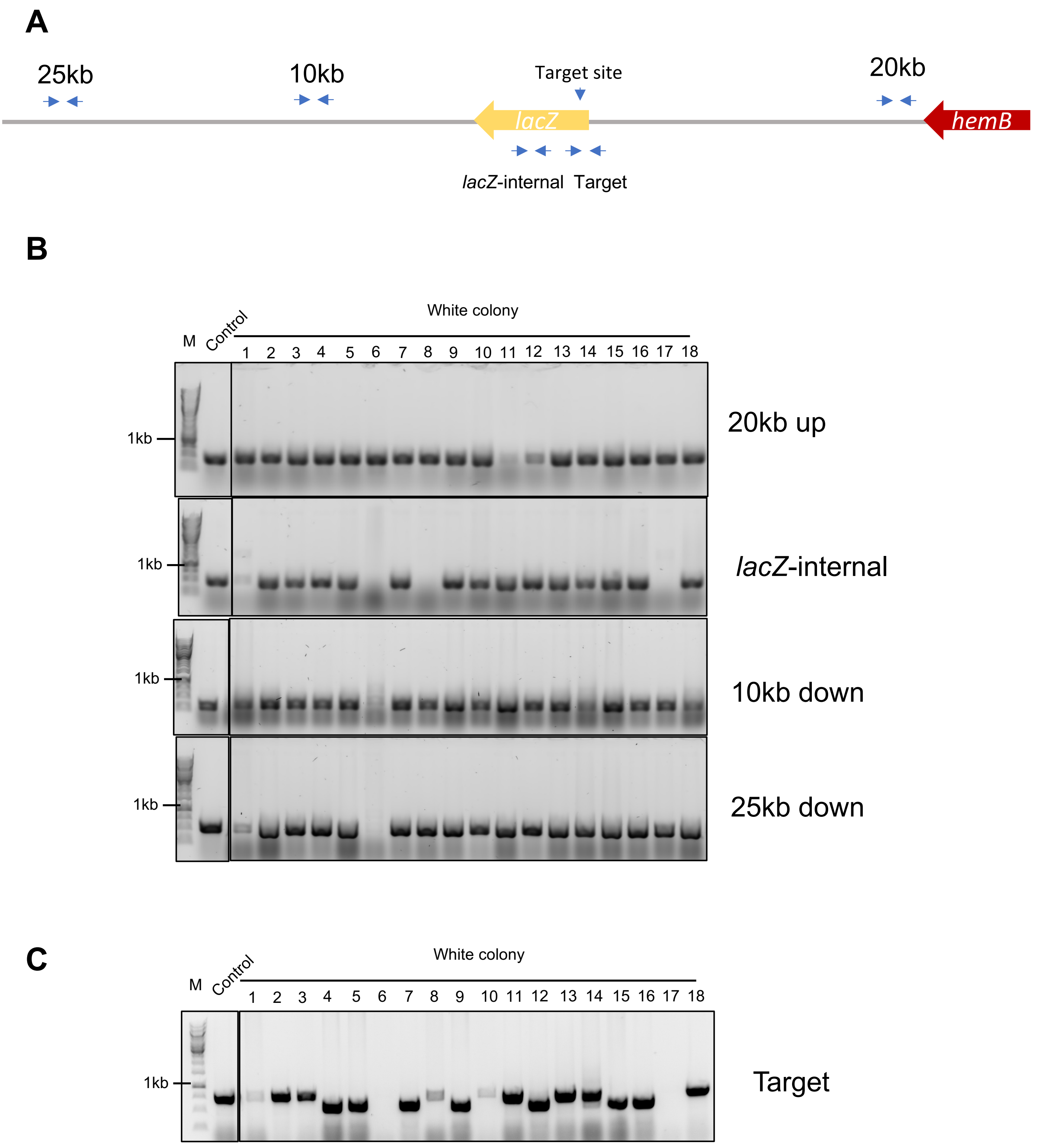

FigureS5. Tiling PCR of type I-G K39A targeting *lacZ*. (A) An overview of location of tiling PCR primers; A pair of small blue arrow represents a set of PCR primers. **(B)** Tiling PCR product from different primers was submitted for electrophoresis on a 0.8% agarose gel. 18 white colonies and 1 blue colony (Control) were assayed. M, marker. **(C)** PCR product with primers that cover the target area was submitted for electrophoresis on a 0.8% agarose gel.

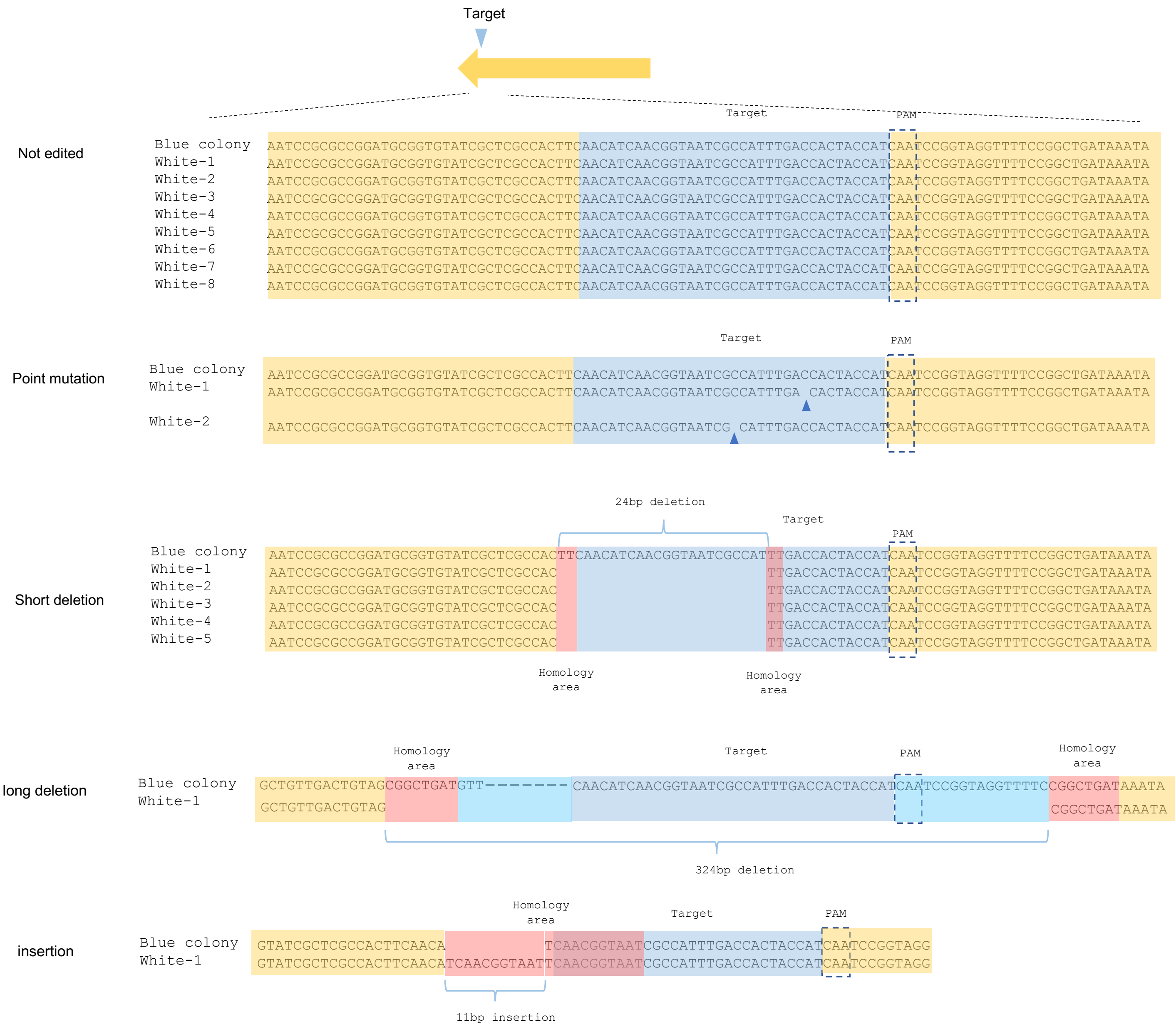

FigureS6. K39A target deletion on alternative *lacZ* site. A different target site on *lacZ* gene produces various editing outcomes; Blue arrow, point mutation site. Homology in red.

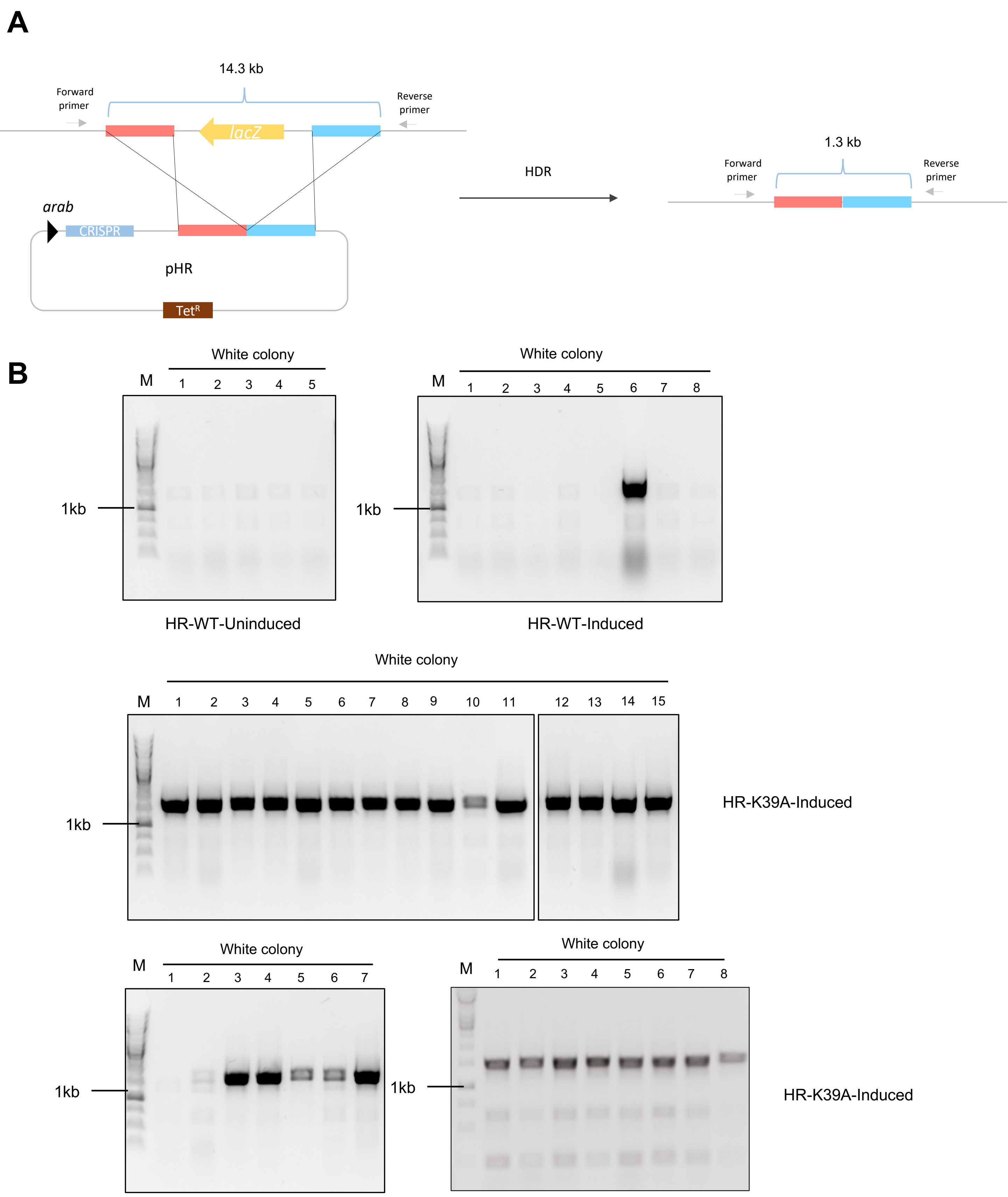

FigureS7. Homologous recombination for repair. (A) A schematic of homologous recombination; Homologous arms in red and blue; Primers for desired HDR verification was shown in grey arrow. **(B)** PCR product with verification primers were submitted for electrophoresis on a 0.8% agarose gel; 1.3kb product was detected, showing desired HDR with donor templates.

Table1. Primers

Name	Squence (5' to 3')	Note
T7toaraBAD-F	gcaggaggaatacccatgggcagcgtcgacatggataaggatatgcacattaatgagatcgtcttgcg	Replace T7 promoter by araBAD promote on pACE-M1
T7toaraBAD-R	ctataacggtcctaaggtagcgacctaggtatcgttatgacaacttgacggctacatcattcac	
lacZ-507-F	TTGTGGAGCGACATCCAGAG	Verification on middle of lacZ
lacZ-507-R	GATGAAGACCAGCCCTTCCC	
lacZ-20kUP-F	GTCTTCCAGGGCGGAAATCA	Verification on 20k upstream lacZ
lacZ-20kUP-R	ATTACCCAGTCGAACCCACG	
lacZ-10kUP-F	CGTCCAGTAACCATGTCGCT	Verification on 10k upstream lacZ
lacZ-10kUP-R	AGAATACCCGGTGCAGAAGC	
lacZ-10kDown-F	ATGTTTTGCTGGTGGATCGC	Verification on 10k downstream lacZ
lacZ-10kDown-R	TATACGAATGCCCCACCACC	
lacZ-25kDown-F	CGCATGGCATCGAATACAGC	Verification on 25k downstream lacZ
lacZ-25kDown-R	GTTCGCACCAGCCAAGAATG	
lacZ-40kDown-F	AACATCATTAGCGGCCCCAG	Verification on 40k downstream lacZ
lacZ-40kDown-R	TTGCCTGGCTCTGGGATTTT	
lacZ-55kDown-F	AACTGGGCTTTCAGTCCTGG	Verification on 55k downstream lacZ
lacZ-55kDown-R	CTTGACGACGGGCAGGTTAT	
lacZ-1-veri-F	GCGAGTGGCAACATGGAAAT	Verification on lacZ-1 target site
lacZ-1-veri-R	TTAGGCACCCCAGGCTTTAC	
lacZ-4-veri-F	CCCCATATGGAAACCGTCGAT	Verification on lacZ-4 target site
lacZ-4-veri-R	TCTGACCACCAGCGAAATGG	
HR-5kdown-F	GGCTCATATGCCGCGCATTCCTCCAA	For homologous arm 5k downstream of lacZ
HR-5kdown-R	CAGCCTCGAGGAGCTGGAGGCAATTCCTTT	
HR-5kUP-F	GCTCCTCGAGAAACCGTTGTCCTGCTGCAT	For homologous arm 5k uptream of lacZ
HR-5kUP-R	gcagcctaggCCCCCAGACAATCAGGGTTT	
lacZ-HRveri-F	GACTGGGTTACAGCGAGCTT	Verification on HDR product
lacZ-HRveri-R	TTAAGGGCGTGCGAGGAAAT	
Cas3 K39A-F	ACTGGGG**GCA**ACCGCGGCCGTGACGC	Cas3 mutagenesis
Cas3 K39A-R	CCTACAGGGATGGCCTGACCCC**CGT**TGGCG	
Cas3 D164A-F	GGTTTTC**GCT**GAGGTACAGTTGATGGGTCCAGG	Cas3 mutagenesis
Cas3 D164A-R	CTGTACCTC**AGC**GAAAACCCATAATGCATCGTTGTGC	

Table2. oligonucleotides

Name	Squence (5' to 3')	Note
Spacer-lacZ-1-T	AAGCACCGTAATGGGATAGGTCACGTTGGTGTAGATGGGC	Spacer targeting lacZ start site
Spacer-lacZ-1-C	TGACGCCCATCTACACCAACGTGACCTATCCCATTACGGT	
Spacer-lacZ-4-T	AAGCATGGTAGTGGTCAAATGGCGATTACCGTTGATGTTG	Spacer targeting lacZ end site
Spacer-lacZ-4-C	TGACCAACATCAACGGTAATCGCCATTTGACCACTACCAT	
Spacer-frmA-T	AAGCTCGTAGTCATTCGGGTTAATGCAGTCGGTAGCACCG	Spacer targeting frmA gene
Spacer-frmA-C	TGACCGGTGCTACCGACTGCATTAACCCGAATGACTACGA	
Spacer-yahK-T	AAGCGAAAACTACTGTGATCACATGACCGGCACCTATAAC	Spacer targeting yahK gene
Spacer-yahK-C	TGACGTTATAGGTGCCGGTCATGTGATCACAGTAGTTTTC	
Tsu-BpiI-rep-T-5'	catggATCGACTTTTCTGCGAGGGCCGTCATCCGCGGCATTTAGCCGCGGCCTCATTGAAGCgtgtctt	Introduce type I-G repeat sequence with two BpiI site
Tsu-BpiI-rep-T-3'	cgtaccttgaagaccaGTCATCCGCGGCATTTAGCCGCGGCCTCATTGAAGCGCCGAATCTCg	
Tsu-BpiI-rep-C-3'	tcgacGAGATTCGGCGCTTCAATGAGGCCGCGGCTAAATGCCGCGGATGACtggtcttcaagg	
Tsu-BpiI-rep-C-5'	tacgaagacacGCTTCAATGAGGCCGCGGCTAAATGCCGCGGATGACGGCCCTCGCAGAAAAGTCGATc	

Table3. Colony counts

	Figure 4A CFU/ml in log						Figure 4B white colony number/ml					
	Uniduced			Induced			Uniduced			Induced		
WT-EV	5.26	5.30	5.08	5.16	5.27	5.05	0	0	0	0	0	0
WT-lacZ	3.57	3.37	3.22	3.60	3.34	3.15	180	130	40	510	410	150
	Figure 4A CFU/ml in log						Figure 4B white colony number/ml					
	Uniduced			Induced			Uniduced			Induced		
K39A-EV	5.35	5.33	5.31	5.30	5.37	5.28	0	0	0	0	0	0
K39A-lacZ	5.36	5.30	5.27	3.59	3.61	3.31	0	0	0	400	550	440
	Figure 5B CFU/ml in log						Figure 5C white colony number/ml					
	Uniduced			Induced			Uniduced			Induced		
WT-EV	5.26	5.30	5.08	5.16	5.27	5.05	0	0	0	0	0	0
WT-HR	3.41	3.13	2.95	3.33	3.01	2.89	50	60	30	160	120	40
K39A-EV	5.35	5.33	5.31	5.30	5.37	5.28	0	0	0	0	0	0
K39A-HR	5.21	5.35	5.25	3.81	3.76	3.75	0	0	0	2000	1800	1970
	Figure S2A CFU/ml in log						Figure S2B white colony number/ml					
	Uniduced			Induced			Uniduced			Induced		
EV	5.26	5.30	5.08	5.16	5.27	5.05	0	0	0	0	0	0
lacZ	3.57	3.37	3.22	3.60	3.34	3.15	180	130	50	510	410	120
frmA	3.40	3.40	3.18	3.40	3.36	3.05	10	0	0	30	10	0
yahK-1	3.35	3.33	3.25	3.39	3.30	3.14	260	120	80	440	330	90
yahK-2	3.65	3.59	3.59	3.47	3.30	3.19	10	20	10	420	270	100

Table4. Plasmids

Name	Description
pACE-M1	Used for plasmid challenge assay and phage challenge assay. csb2, cas7 and cas8g genes included.
pCDF	Used for plasmid challenge assay and phage challenge assay. CRISPR array targeting tetR or phage lpa included.
pRAT-Duet	Used for plasmid challenge assay and phage challenge assay control. Lacking cas3.
pRAT-Cas3	Used for plasmid challenge assay and phage challenge assay. cas3 included.
pM2	Used for genome targeting. cas3, csb2, cas7 and cas8g genes included.
pSPACER	Used for genome targeting. CRISPR array included.
pHR	Used for genome targeting. CRISPR array and homologous arms included.
